# Arterial spin labeling using spatio‐temporal encoding readout for robust perfusion imaging in inhomogenous magnetic fields

**DOI:** 10.1002/mrm.29506

**Published:** 2022-11-24

**Authors:** Suzanne L. Franklin, Megan Schuurmans, Martins Otikovs, Pim T. S. Borman, Matthias J. P. van Osch, Clemens Bos

**Affiliations:** ^1^ Center for Image Sciences University Medical Center Utrecht Utrecht The Netherlands; ^2^ C.J. Gorter Center for High Field MRI, Department of Radiology Leiden University Medical Center Leiden The Netherlands; ^3^ Department of Chemical and Biological Physics Weizmann Institute of Science Rehovot Israel; ^4^ Department of Radiotherapy University Medical Center Utrecht Utrecht The Netherlands; ^5^ Leiden Institute for Brain and Cognition Leiden University Leiden The Netherlands

**Keywords:** arterial spin labeling, ASL, implant, robust, Spatio‐temporal encoding, SPEN

## Abstract

**Purpose:**

To evaluate the feasibility of spatio‐temporal encoding (SPEN) readout for pseudo‐continuous ASL (pCASL) in brain, and its robustness to susceptibility artifacts as introduced by aneurysm clips.

**Methods:**

A 2D self‐refocused T_2_*‐compensated hybrid SPEN scheme, with super‐resolution reconstruction was implemented on a 1.5T Philips system. *Q* (=BW_chirp_*T_chirp_) was varied and, the aneurysm clip‐induced artifact was evaluated in phantom (label‐images) as well as in vivo (perfusion‐weighted signal (PWS)‐maps and temporal SNR (tSNR)). In vivo results were compared to gradient‐echo EPI (GE‐EPI) and spin‐echo EPI (SE‐EPI). The dependence of tSNR on TR was evaluated separately for SPEN and SE‐EPI. SPEN with *Q* ˜ 75 encodes with the same off‐resonance robustness as EPI.

**Results:**

The clip‐induced artifact with SPEN decreased with increase in Q, and was smaller compared to SE‐EPI and GE‐EPI in vivo. tSNR decreased with Q and the tSNR of GE‐EPI and SE‐EPI corresponded to SPEN with a Q‐value of approximately ˜85 and ˜108, respectively. In addition, SPEN perfusion images showed a higher tSNR (*p* < 0.05) for TR = 4000 ms compared to TR = 2100 ms, while SE‐EPI did not. tSNR remained relatively stable when the time between SPEN‐excitation and start of the next labeling‐module was more than ˜1000 ms.

**Conclusion:**

Feasibility of combining SPEN with pCASL imaging was demonstrated, enabling cerebral perfusion measurements with a higher robustness to field inhomogeneity (*Q* > 75) compared to SE‐EPI and GE‐EPI. However, the SPEN chirp‐pulse saturates incoming blood, thereby reducing pCASL labeling efficiency of the next acquisition for short TRs. Future developments are needed to enable 3D scanning.

## 1 INTRODUCTION

Arterial spin labeling (ASL) is a non‐contrast enhanced perfusion method, used for numerous applications both in brain^1^, ^2^ as well as body.^3^, ^4^, ^5^, ^6^, ^7^ ASL is often used in combination with single‐shot acquisition techniques, such as EPI, because of their time efficiency, and motion insensitivity, which is especially important for body applications.^4^, ^6^ However, EPI is especially prone to distortion and chemical shift artifacts in the phase‐encoding (PE) direction, due to weak gradients. Many body applications, such as imaging of kidney,^4^ breast,^8^ but also brain applications, e.g., in patients with implants^9^ or cerebral hemorrhages,^10^, ^11^ are characterized by magnetic field distortions, complicating the application of ASL.

Spatio‐temporal encoding (SPEN) has been introduced in 2005^12^ as an alternative to EPI and can provide increased robustness to magnetic field inhomogeneity in similar acquisition time.^13^ In functional^14^ as well as diffusion‐weighted^15^ MRI, SPEN was shown to be superior to EPI in areas associated with susceptibility‐effects, such as the (orbito)frontal cortex of the brain,^14^, ^16^ breast^15^ and in the vicinity of metal implants.^17^, ^18^

**FIGURE 1:**
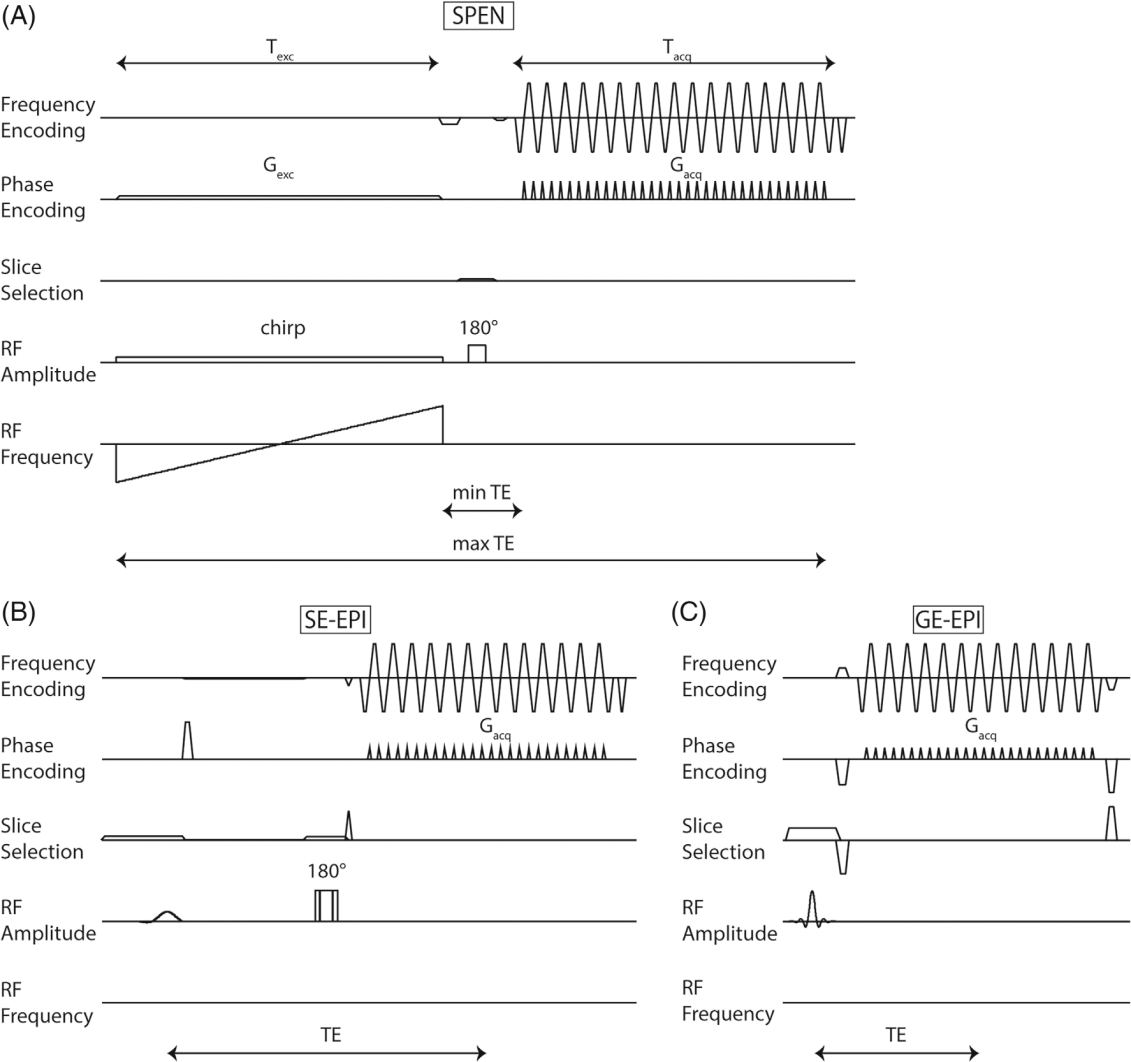
Scaled sequence diagrams for the SPEN (*Q* = 125) (A), SE‐EPI (B), and GE‐EPI readout (C), used in this study. The acquisition gradient (G_acq_) in phase‐encoding direction is larger for SPEN compared to GE‐EPI and SE‐EPI, leading to a larger robustness for magnetic field offsets. For SPEN, the frequency‐swept chirp‐pulse is used as excitation pulse. The chirp‐pulse in combination with the 180‐degree refocusing pulse, results in sequentially generated echoes in time, related to different parts of the FOV. This leads to a varying T_2_‐weighting over the FOV: spins that are first excited will have a maximum TE, while spins that are excited last will have felt a minimum TE. The excitation time (T_exc_) was chosen equal to the acquisition time (T_acq_), resulting in full‐T_2_* refocusing

SPEN uses a frequency‐swept (chirp) pulse in combination with a gradient, which leads to a local excitation, that excites the FOV over time. This results in a linear relationship between spatial location and time, and thus no Fourier transformation is required in the direction of this excitation gradient. Super‐resolution (SR) reconstruction is performed to enable short scan times without compromising on resolution or specific absorption rate (SAR), compared to EPI.^19^, ^20^ SPEN is characterized by a stronger mean acquisition gradient (G_acq_) as compared to EPI (Figure 1), which reduces image distortion (displacement [d]) as a result of susceptibility‐effects: $d=\frac{\Delta B_{0}}{G_{\mathrm{acq}}}$ . Sensitivity to susceptibility‐effects can further be reduced by full T_2_*‐refocusing of all data, when using appropriate timings. However, the frequency swept pulse in SPEN could potentially saturate incoming blood (i.e., blood below the labeling plane), which would in turn reduce labeling efficiency during the following labeling module, thereby necessitating a longer TR to preserve temporal SNR (tSNR).

In this technical note, the feasibility of combining SPEN with pseudo‐continuous ASL (pCASL) for cerebral perfusion imaging was explored: (1) The behavior of SPEN as a function of *Q* (=BW_chirp_*T_chirp_ [−]), which determines the robustness to susceptibility‐effects and SNR, is investigated in the presence of an aneurysm clip, in a phantom, and (2) in vivo. (3) The perfusion‐weighted signal (PWS)‐map, in the presence of an aneurysm clip, is compared between SPEN, gradient‐echo EPI (GE‐EPI), and spin‐echo EPI (SE‐EPI). (4) The effect of TR on the temporal SNR (tSNR) of the PWS‐images for SPEN and SE‐EPI is investigated.

## 2 METHODS

### 2.1 Data acquisition

This study was approved by the local ethics committee (METC Utrecht, Protocol 15‐466) Informed consent was obtained from all participants. Nine healthy volunteers (six females, 26–32 years), were scanned on an 1.5T Philips Achieva scanner (Philips) using an eight‐channel head coil.

A 2D self‐refocused T_2_*‐compensated hybrid SPEN scheme was used, where SPEN encoding in the original PE direction, i.e., anterior–posterior, was combined with conventional k‐space encoding in the frequency‐direction.^13^ The sequence included; a 90‐degree chirp‐pulse with a duration equal to the acquisition duration (T_chirp_ = T_acq_ = 20.4 ms); and a 180‐degree spin‐echo pulse, positioned symmetrically in between the chirp‐pulse and start of acquisition, to ensure complete T_2_*‐refocusing (Figure 1). Depending on Q, we adjusted B_1_, G_exc_ the excitation gradient under the chirp pulse, and G_acq_, the time averaged gradient in SPEN direction during data acquisition according to: $B_{1}=0.27*\frac{\sqrt{Q}}{\gamma T_{\mathrm{exc}}},$ ^21^ $G_{\mathrm{exc}}=\frac{Q}{\gamma L_{\mathrm{pe}}T_{\mathrm{exc}}}$, and $G_{\mathrm{acq}}=\frac{Q}{\gamma L_{\mathrm{pe}}T_{\mathrm{acq}}}$ (*L*_pe_ = FOV in SPEN‐direction), see also Supporting Information on the relation between *Q* and mean gradient strength.

SPEN was acquired with an FOV of 200 × 200 mm, slice thickness of 10 mm, voxel size of 3.1 mm in read‐direction, and $\Delta y_{\text{SPEN}}=\frac{L_{\mathrm{pe}}}{N_{\text{SPEN}}}$ = 5.7 mm, i.e., 35 apex locations in the SPEN‐direction. $\Delta y_{\text{SPEN}}\;$ corresponds to the shift of the quadratic phase‐profile between readout lines. This results in a TE range of 5–45 ms over the FOV. All scans were acquired with slope sampling turned off, and all in vivo scans were acquired with Spectral Presaturation with Inversion Recovery (SPIR) fat suppression.

For all in vivo scans, separate M_0_‐images were acquired with the same readout and *Q*‐value setting, by turning ASL labeling off and TR = 2000 ms, resulting in durations of 8 s.

#### 2.1.1 Experiment 1

A phantom and aneurysm clip (titanium, 2 cm), see Figure S1, were placed in water doped with 0.4 mM Gd. SPEN was acquired with a TR of 4000 ms and Q‐values of; 50, 75, 100, 12, 150, and 200, each corresponding to an off‐resonance robustness (BW/mm) of 12.3, 18.3, 24.7, 30.7, 36.6, and 49.4 Hz/mm. This series was acquired with and without SPIR.

#### 2.1.2 Experiment 2

Two healthy volunteers were scanned with the aneurysm clip, positioned on the forehead and fixated by tape. SPEN was acquired with *Q*‐values; 50, 75, 100, 125, and 150. In volunteer 2, an image with *Q* = 175 was additionally acquired, corresponding to 43.0 Hz/mm.

SE‐EPI and GE‐EPI‐scans were acquired with the same FOV, slice thickness as SPEN, acquired voxel size of 3.1 mm × 3.0 mm, SENSE factor of 2.3, and TR of 4000 ms. The TE was 17, and 9.1 ms, for SE‐EPI and GE‐EPI, respectively. These settings correspond to a BW/mm of 18.7 Hz/mm. So, the off‐resonance robustness, and thus PE/SPEN bandwidth, of SE−/GE‐EPI is similar to SPEN with *Q* ˜ 75. Note that, according to Ref.,^22^ in principle, SR reconstruction would allow to reconstruct SPEN with *Q* ≥ 75 at a resolution at least as high as EPI.^22^

These scans were acquired with pCASL labeling duration of 1800 ms, background suppression, and post‐label delay (PLD) of 1800 ms and 30 repetitions. Background suppression included presaturation of the imaging stack just before start of labeling, and two non‐selective hyperbolic secant pulses at 2250 ms and 3150 ms after presaturation. The total scan duration was 4 min 16 s for SPEN and SE‐EPI, and 4 min 8 s for GE‐EPI‐scans.

In addition, in volunteer 2, a T_1_‐weighted scan without the aneurysm clip was acquired, using multi‐slice single‐shot SE‐EPI, FOV of 230 × 183 mm, voxel sizes of 0.9 × 1.12 × 5 mm, 21 slices, and TR/TE of 570 ms/15 ms.

#### 2.1.3 Experiment 3

In the same volunteers of experiment 1, additional pCASL‐scans with SPEN, SE‐EPI, and GE‐EPI readout were acquired without an aneurysm clip positioned on the forehead, using the same settings. SPEN was acquired with *Q* = 125 and *Q* = 175 for volunteer 1 and 2, respectively.

#### 2.1.4 Experiment 4

Six volunteers were scanned with a series of SPEN and SE‐EPI‐scans, where TR was varied according to: 2100, 2300, 2600, 3000, 3500, and 4000 ms. SPEN‐scans were acquired with *Q* = 50 (12.4 Hz/mm), to have a favorable SNR of ASL when measuring tSNR as a function of TR. For the SE‐EPI scans, the same settings were used as described in experiment 2. Scans were acquired with a labeling duration of 1000 ms, PLD of 1000 ms, and 30 repetitions. Scan durations were the same for SPEN and SE‐EPI, ranging between 2 min 14 s and 4 min 16 s for TRs of 2100 and 4000 ms, respectively. Background suppression was turned off. In addition, a T_1_‐weighted scan was acquired.

### 2.2 Data analysis

K‐space data of the SPEN‐images were exported from the scanner, and pre‐processing, up to (not including) the Fourier‐transformation, of the data was done using ReconFrame (Gyrotools, Zurich, Switzerland). The data were reconstructed following steps described by Seginer et al,^23^ by adapting the algorithm,^24^ where instead of L2‐regularized iterative reconstruction used in reference,^24^ final images were obtained by a multiplication with a SR matrix.^25^

Co‐registration and motion correction was performed using a rigid PCA‐based registration method^26^ using Mevislab (MeVis Medical Solutions, Bremen, Germany). SPEN, SE‐EPI, and GE‐EPI‐scans were all separately co‐registered to the T_1_‐scan. Perfusion‐weighted maps were calculated using:

(1) $$∆S_{i}=\frac{1}{R}\;\sum_{r=1}^{R}\left(C_{r,i}-L_{r,i}\right)$$

(2) $${\mathrm{PWS}}_{i}\left[\%\right]=\frac{∆S_{i}}{M_{0,i}}$$

Where: *C*_*r,i*_ = control‐image, *L*_*r,i*_ = label‐image, *r* = repetitions, *i* = voxels, Δ*S*_*i*_ = subtraction‐image, *M*_0,*i*_ = the scan used to calibrate the ASL‐signal according to the expected blood signal.^2^ Brain masks, used for visualization of the PWS‐maps, were generated using SPM (Wellcome Center for Human Neuroimaging, London, United Kingdom), based on the *M*_0_‐scans acquired without aneurysm clip. GM segmentation was performed on the T_1_‐weighted scans using SPM (using >70% GM contribution).

tSNR maps were generated using:

(3) $${\text{tSNR}}_{i}=\frac{\mu_{∆S,i}}{\sigma_{∆S,i}}$$

where *μ*_Δ*S*,*i*_ = mean ASL‐signal over all repetitions, and *σ*_Δ*S,i*_ = SD over time. The tSNR in GM was calculated by averaging the tSNR in all GM voxels.

#### 2.2.1 Experiment 1

Label‐images were generated for each *Q*‐value.

#### 2.2.2 Experiment 2

Label‐images, *M*_0_‐images, PWS‐maps, and tSNR‐maps were generated for each *Q*‐value. To calculate mean tSNR in GM, the part of the FOV containing the clip‐induced artifact was omitted from the GM‐mask, by taking out a square of 1/3 of FOV. To illustrate the relation between the tSNR in GM of SPEN and SE−/GE‐EPI, the tSNR of the EPI‐images were corrected by a factor Δ*y*SPEN/Δ*y*EPI (Δ*y*EPI = the acquired voxel size of the EPI‐scans). This was done to compensate, in a best‐effort manner, for the smaller voxel sizes with which the EPI images were acquired, while recognizing that this compensation will not be identical to acquisition at larger voxel‐size.

#### 2.2.3 Experiment 3

Label‐images and PWS‐maps were generated for SPEN, SE‐EPI, and GE‐EPI with and without aneurysm clip.

#### 2.2.4 Experiment 4

tSNR maps, and the average tSNR in GM over all volunteers was calculated for each TR, for SPEN and SE‐EPI. A non‐parametric Wilcoxon rank sum test (*α* = 0.05) was used to test whether the tSNR of TR = 2100 ms was significantly different from TR = 4000 ms.

## 3 RESULTS

**FIGURE 2:**
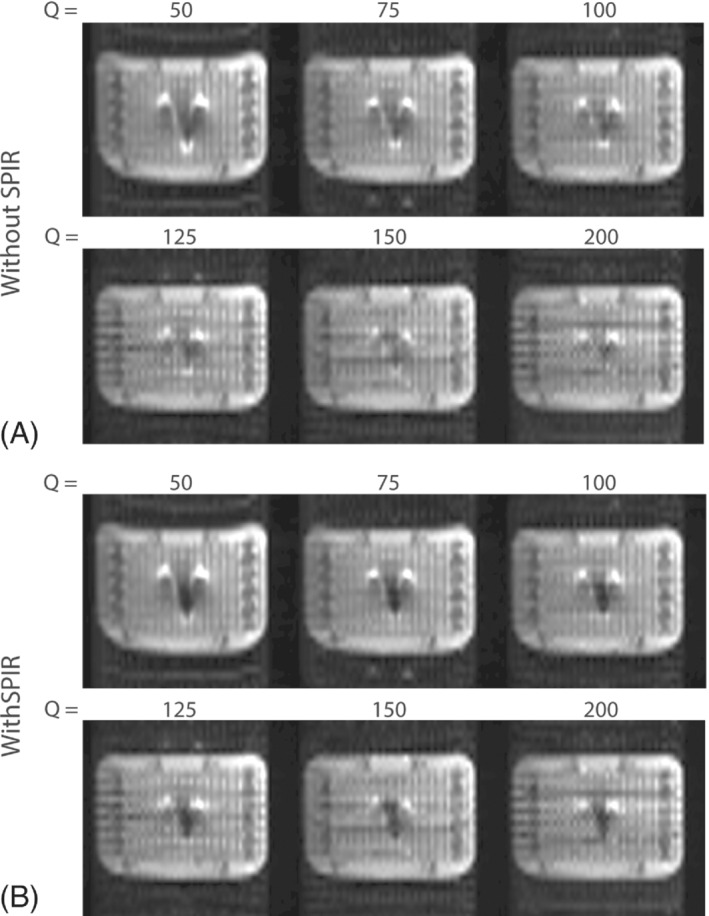
Phantom with aneurysm clip, acquired with a SPEN‐readout at different *Q*‐values (50, 75,  100,  125,  150,  200), without SPIR fat suppression (A), with SPIR fat suppression (B). A, The artifact induced by the aneurysm clip is reduced for higher *Q*‐values. B, This effect becomes less apparent when using SPIR fat suppression. Signal intensity is normalized per *Q*‐value to facilitate comparison of field inhomogeneity effects between the images

A higher Q‐value decreases the aneurysm clip‐induced artifact, as demonstrated in a phantom, see Figure 2A. When using SPIR a larger clip‐induced artifact remains, see Figure 2B. In both cases, additional through‐plane slice‐selection effects lead to signal voids near the aneurysm clip, see Figure S1. For practical reasons, all in vivo data were acquired using $\Delta y_{\text{SPEN}}$ 5.7 mm, but using identical Δ*y*_SPEN_ and Δ*y*_EPI_ shows the resolution potential of SPEN in the phantom, see Figure S2. Also, in vivo SPIR was deemed necessary; thus, the effect of Q on the clip‐induced artifact size is reduced, see Figure S2.

**FIGURE 3:**
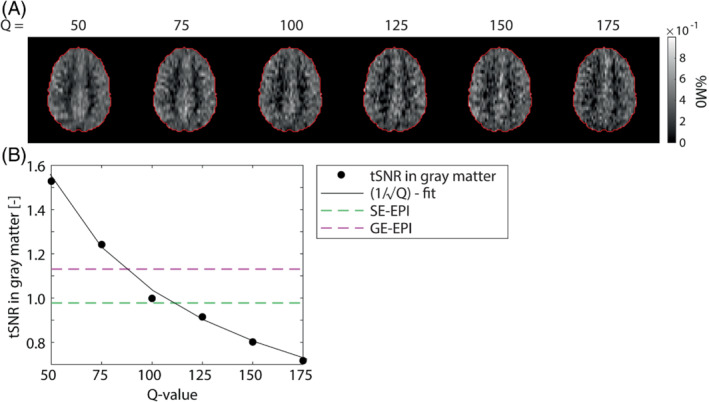
A, SPEN perfusion‐weighted signal (PWS)‐images at *Q*‐values between 50 and 175. B, Mean tSNR in gray matter as a function of Q‐value. The data are fitted with a 1/√Q – function. For illustration purposes, the tSNR‐value of SE‐EPI and GE‐EPI were compensated in a best‐effort manner for the differences in acquisition voxel size, shown in green and magenta, respectively.

The label‐images show some ghost artifacts for higher *Q*‐values (>±125), indicated by black arrowheads in Figure S3A. The tSNR of the PWS‐images shows a 1/√Q‐relation. Including the factor that attempts to compensate for the difference in acquisition voxel size between SPEN and SE−/GE‐EPI, the tSNR of SE−/GE‐EPI is associated with the tSNR of SPEN with *Q* = 108/85 respectively, see Figure 3.

**FIGURE 4:**
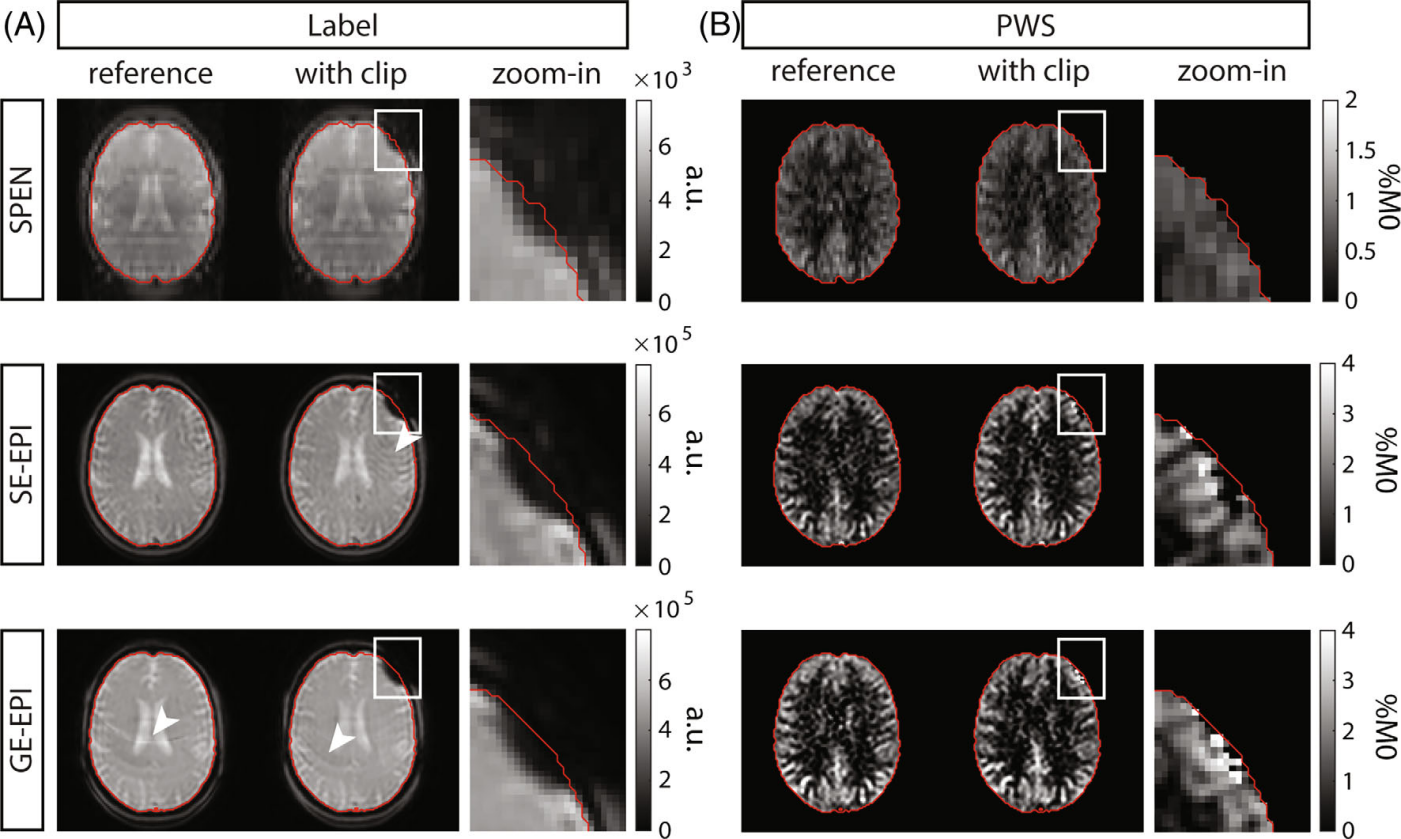
Comparing the effect of an aneurysm clip on SPEN with *Q* = 125, SE‐EPI and GE‐EPI. The pCASL label images (A), and perfusion‐weighted signal (PWS) maps (B), without (reference) and with (with clip) aneurysm clip. The last column shows a zoom‐in of the artifact area, of which the exact location is depicted by the white square. The clip‐induced artifact is smaller for SPEN compared to SE‐EPI and GE‐EPI. In addition, SE‐EPI showed a Gibbs ringing artifact anterior to the aneurysm clip, and GE‐EPI showed ghost artifacts, both indicated by white arrows

The aneurysm clip‐induced artifact is smaller for the SPEN compared to SE−/GE‐EPI, and SE‐EPI and GE‐EPI show additional artifacts, see Figure 4. In the PWS‐maps, small subtraction artifacts can be observed at the location of the aneurysm clip for SE‐EPI and GE‐EPI, which are not visible for SPEN, see Figure 4.

**FIGURE 5:**
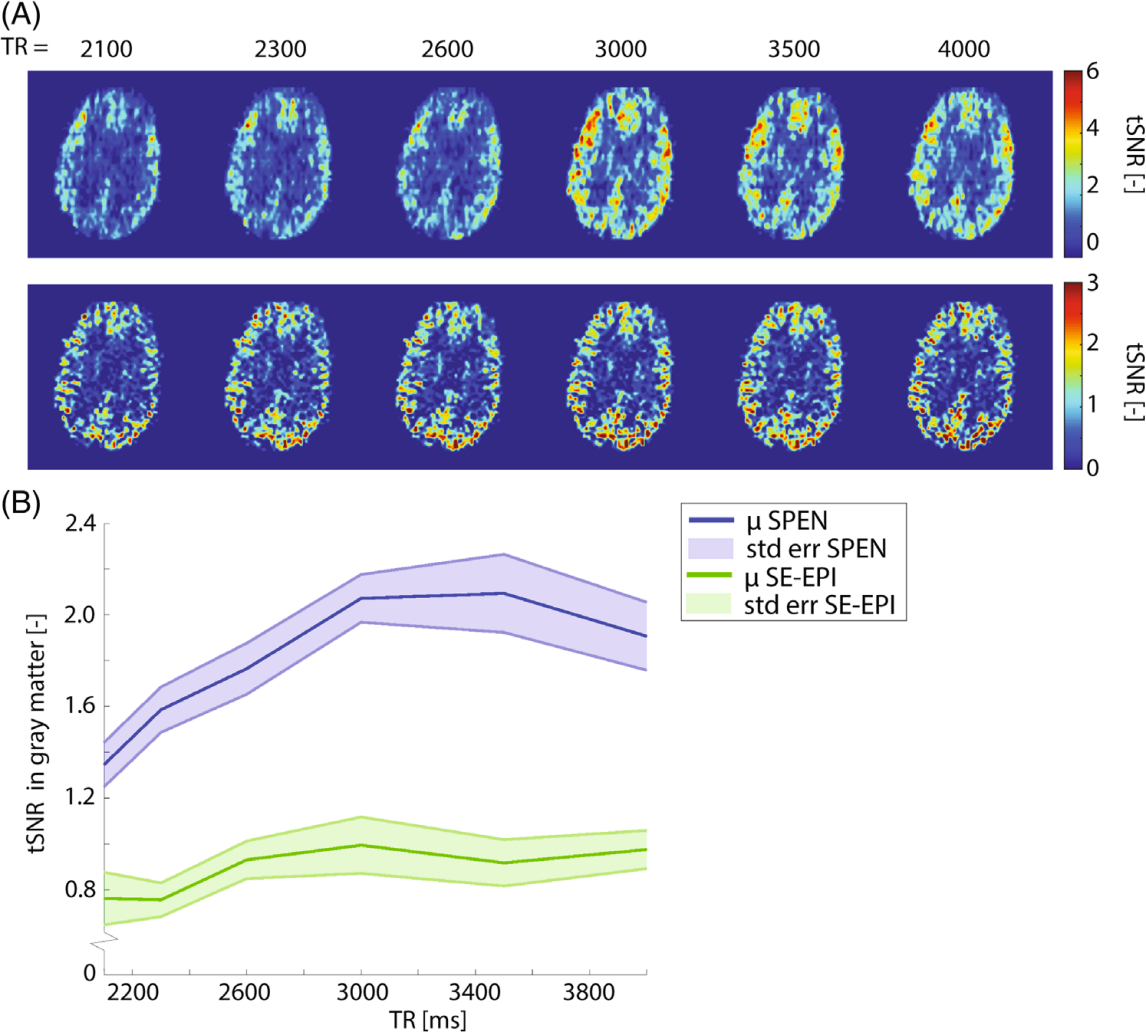
A, Effect of TR on SPEN and SE‐EPI for a representative volunteer. B, Mean (μ) tSNR in gray matter over all volunteers as a function of TR, for SPEN (blue) and SE‐EPI (green). The shaded area represents the standard error (std err) of tSNR

tSNR increases noticeably with TR for SPEN, see Figure 5A. This trend was confirmed by quantitative evaluation of tSNR over all volunteers, see Figure 5B. tSNR was significantly different between the TR = 2100 ms and TR = 4000 ms for SPEN (*p* = 0.026, median difference = 0.551), and not for SE‐EPI (*p* = 0.240, median difference = 0.207). No direct comparison of tSNR values with EPI should be made for this experiment since a relatively low *Q*‐value (*Q* = 50) was used for SPEN.

## 4 DISCUSSION

Results showed the feasibility of SPEN in pCASL imaging, enabling cerebral perfusion measurements with a higher robustness to susceptibility‐effects compared to SE−/GE‐EPI, by using the appropriate *Q*‐value. The 1/√*Q*‐relation between tSNR of SPEN and *Q*‐value, as previously derived in reference,^20^ was confirmed. Furthermore, a dependence on TR was observed for pCASL‐images acquired with SPEN.

SPEN enables robust imaging via two mechanisms: SPEN has a stronger G_acq_, resulting in reduced image distortions as a result of susceptibility‐effects.^13^ Second, SPEN was acquired in full‐refocusing mode, meaning that every acquired point was close to being completely refocused (instead of just the center of k‐space), reducing signal loss due to T_2_*.^13^ Experiment 1 and 2 showed that SPEN had a smaller clip‐induced artifact than SE‐EPI and GE‐EPI, for all Q‐values, confirming the higher robustness to field‐inhomogeneity of SPEN. Similar as was observed in the vicinity of metallic implants.^18^ Increasing Q results in stronger acquisition gradients,^25^ so smaller distortions as a result of field inhomogeneities are expected. Results showed a reduction in the clip‐induced artifact for higher *Q*‐values, evidencing a higher robustness to susceptibility‐effects. In vivo, the clip‐induced artifact remained relatively stable over *Q*. This is likely mainly due to the suppression of water signal, caused by sensitivity to off‐resonance of SPIR, as well as through‐plane effects caused by distorted excitation profiles^27^ due to limited bandwidth of the slice‐selective refocusing pulse, both demonstrated in the phantom data.

tSNR in GM of SPEN dropped according to 1/√*Q*, confirming the theoretical derivation by Ben‐Eliezer et al.^20^ With Q the quadratic phase‐profile narrows, resulting in a lower SNR. In the illustration of tSNR of SPEN and SE−/GE‐EPI (Figure 3) a factor was used to try to compensate for acquisition voxel size differences. Note, however that there is still an ongoing discussion on the spatial sensitivity in SPEN imaging as compared to that in Fourier encoded imaging, making such compensations only approximations.^28^ Using the current settings, the tSNR in GM of SE‐EPI and GE‐EPI is associated with that of SPEN using a *Q*‐value of approximately ˜85 and ˜108, respectively, based on the 1/√*Q*‐curve. Importantly, the G_acq_ of SPEN is higher than of SE−/GE‐EPI, at *Q* = 85 and 108, since the BW/mm of SE‐EPI and GE‐EPI corresponds to *Q* ˜ 75. Meaning that if *Q* ≥ 75 and *Q* ≤ 108 (SE‐EPI) or Q85 ≤ (GE‐EPI) SPEN can produce perfusion images with improved robustness to field homogeneities at little cost to tSNR.

For *Q* ≥ ±125, ghost artifacts were observed. They are a result of the SR reconstruction and are akin to Nyquist ghosts seen in EPI readouts.^23^ These artifacts can be prevented by acquiring more points in the SPEN direction or by using alternative post‐processing techniques.^29^ However, the former would inevitably increase ASL signal loss due to T_2_‐decay.The longest TE in SPEN is equal to T_exc_ + T_acq_ + TE_min_ (time in between the chirp‐pulse and the start of acquisition, see Figure 1). So the requirement to limit TE results in constraints on the maximum achievable FOV and acquisition matrix. Multi‐shot 2D SPEN^18^ could be an alternative, but in line with most common body ASL protocols,^30^ we opted to stick to a single‐shot readout. In contrast to SPEN, voxel sizes of SE−/GE‐EPI could be chosen smaller without a large penalty on TE, partly due to the use of parallel imaging. Recent developments exploring parallel imaging methods for SPEN have been introduced, e.g., by simultaneously acquiring separate parts of the FOV using multi‐band chirp pulses^15^, ^19^, ^31^ or by acquiring low‐resolution images and employing k‐space interpolations, based on multiple‐receivers, to increase resolution.^22^

Experiment 4 furthermore demonstrated that, when using a short pCASL labeling duration and PLD to become sensitive to the start of the bolus, a higher tSNR for SPEN is observed at longer TRs, which was not the case for SE‐EPI. Background suppression was turned off to prevent additional effects on the signal‐evolution. The tSNR of SPEN followed a recovery‐like pattern over TR, approaching an equilibrium for long TR (>3000 ms). The results are in accordance with the hypothesis that the chirp‐pulse of the previous SPEN readout saturates incoming blood, anterior to the labeling slab, thereby reducing pCASL labeling efficiency. Increasing the TR, and thus the time between end of the SPEN‐readout and start of labeling, allows the inflow of fresh blood, removing this effect. From a TR of approximately 3000 ms, i.e., a pause of 920 ms, this effect was negligible, showing that sufficient fresh inflow together with relaxation can mitigate the saturation effects, albeit at the cost of imaging time.

### 4.1 Limitations

All scans were performed at 1.5T, even though most ASL is performed at 3T. As ASL is increasingly being used for body applications, studies will more frequently be done at 1.5T. The advantages of smaller B_0_‐ and physiological artifacts might outweigh the SNR‐loss and shorter T_1_ of blood. In addition, when scanning patients with implants the lower field strength might be advantageous. Still, it would be interesting to investigate the performance of SPEN at 3T in future studies.

A single‐slice 2D SPEN sequence was used. The use of a frequency swept pulse is inherently challenging to combine with multi‐slice imaging. Various approaches for multi‐slice and 3D imaging have been published, while SAR and SNR limitations are considered,^19^, ^32^, ^33^, ^34^ providing an interesting future direction to enable whole‐brain ASL with SPEN in an acceptable scan time.

In the comparison between SPEN, and SE−/GE‐EPI, a relatively high *Q*‐value (=125) was used for SPEN, meaning that the encoded resolution was higher for the SPEN than the EPI readouts. Refinements to the super resolution reconstruction,^22^ which were not implemented in the current study, may be used to preserve the smaller clip‐induced artifact while maintaining resolution.

The tSNR relation between SPEN and SE−/GE‐EPI should be interpreted carefully as there is still a debate on the spatial selectivity of SPEN measurements as a function of the acquisition parameters. Using a definition which is unfairly small for SPEN could lead to a unjustified favorable tSNR for SPEN.

Results were not reported as quantitative CBF‐values, because this would be of limited additional value over the PWS‐values that were normalized by M0. Performing this quantification would only add scaling factors. However, an additional correction for T2(*) might be required. The correction for T2(*) in SPEN is, however, not trivial because of the variation in TE over the FOV. This should be addressed in future studies.

In the volunteer studies (Figure 4), we invariably observed lower PWS‐values in SPEN compared to EPI, some of which we could to attribute to saturation effects in our TR‐experiment, however, a more detailed analysis of this effect is warranted. This dependence on TR would also affect the quantitative CBF values calculated based on these PWS data.

Next to EPI, other readouts are used for ASL. In brain, 3D segmented methods, such as relaxation enhancement (RARE) stack of spirals or GRASE are recommended,^2^ which are more robust to field inhomogeneity than EPI.^35^, ^36^ Also in body ASL, RARE with various approaches to shorten acquisition time^37^, ^38^ and single‐shot 3D GRASE have been introduced.^39^ Phase‐encoded xSPEN has previously been shown to have similar resolution and SNR as RARE, but in half the scan time.^40^ Future studies making a direct comparison between SPEN and RARE approaches are necessary. The current study focused on brain measurements in the presence of an implant, as a first attempt for SPEN‐ASL. For future studies, it would be interesting to combine SPEN with ASL in body applications.

## 5 CONCLUSIONS

The study showed the feasibility of SPEN‐ASL and demonstrated the performance in the vicinity of an aneurysm clip implant. In addition, considerations have been raised concerning a minimal pause of ˜1000 ms between the SPEN‐excitation pulse and the start of the next labeling module. ASL‐images acquired with SPEN benefit from a higher robustness to field inhomogeneity. However, developments need to be made to increase acquisition speed, to allow smaller voxel sizes in combination with larger FOVs, and enable 3D‐scanning.

## Supporting information


**FIGURE S1.** (A) Titanium aneurysm clip (2 cm), used in the phantom and in‐vivo scans, placed on the phantom; a plastic grid with 8.0 mm periodicity. (B) Reformat demonstrating the distortion of the slice profile, indicated by white arrowheads, of the phantom as shown in (A). Images were to demonstrate the through‐plane effect
**FIGURE S2**. Images of a phantom grid with aneurysm clip, showing SE‐EPI (A, D), GE‐EPI (B, E) and SPEN (C, F) at comparable acquired base resolutions $\Delta y_{\text{SPEN}}$ and $\Delta y_{\mathrm{EPI}}$. SPEN images were acquired using *Q* = 75. Top row: Δ*y* = 5.7 mm; bottom row Δ*y* = 3.1 mm. To achieve 3.1 mm base resolution with SPEN, the FOV was reduced to 140 mm. Images demonstrate the improved resolution when a closer linespacing $\Delta y_{\text{SPEN}}$ is used in acquisition.
**FIGURE S3**. SPEN label‐images for *Q*‐values ranging between 50 and 175. (A) SPEN Label images, edge artifacts are visible for higher *Q*‐values as a result of too few measurement points, indicated by black arrowheads (B) Intensity profiles along the green line in A, for all *Q*‐values, using a spline interpolation with a factor of 2. The intensity step remains roughly at the same location for the different *Q*‐values.
