# Supplementary material for: Arterial spin labeling using spatio‐temporal encoding readout for robust perfusion imaging in inhomogenous magnetic fields
Source: Magn Reson Med. 2022 Nov 24;89(3):1092–101. doi: 10.1002/mrm.29506 (PMC10099794; doi:10.1002/mrm.29506)
Supplement: Supplementary file 1 — FIGURE S1. (A) Titanium aneurysm clip (2 cm), used in the phantom and in‐vivo scans, placed on the phantom; a plastic grid with 8.0 mm periodicity. (B) Reformat demonstrating the distortion of the slice profile, indicated by white arrowheads, of the phantom as shown in (A). Images were to demonstrate the through‐plane effect FIGURE S2. Images of a phantom grid with aneurysm clip, showing SE‐EPI (A, D), GE‐EPI (B, E) and SPEN (C, F) at comparable acquired base resolutions ΔySPEN and ΔyEPI. SPEN images were acquired using Q = 75. Top row: Δy = 5.7 mm; bottom row Δy = 3.1 mm. To achieve 3.1 mm base resolution with SPEN, the FOV was reduced to 140 mm. Images demonstrate the improved resolution when a closer linespacing ΔySPEN is used in acquisition. FIGURE S3. SPEN label‐images for Q‐values ranging between 50 and 175. (A) SPEN Label images, edge artifacts are visible for higher Q‐values as a result of too few measurement points, indicated by black arrowheads (B) Intensity profiles along the green line in A, for all Q‐values, using a spline interpolation with a factor of 2. The intensity step remains roughly at the same location for the different Q‐values. [file MRM-89-1092-s001.docx]

**Supporting Information Figures**


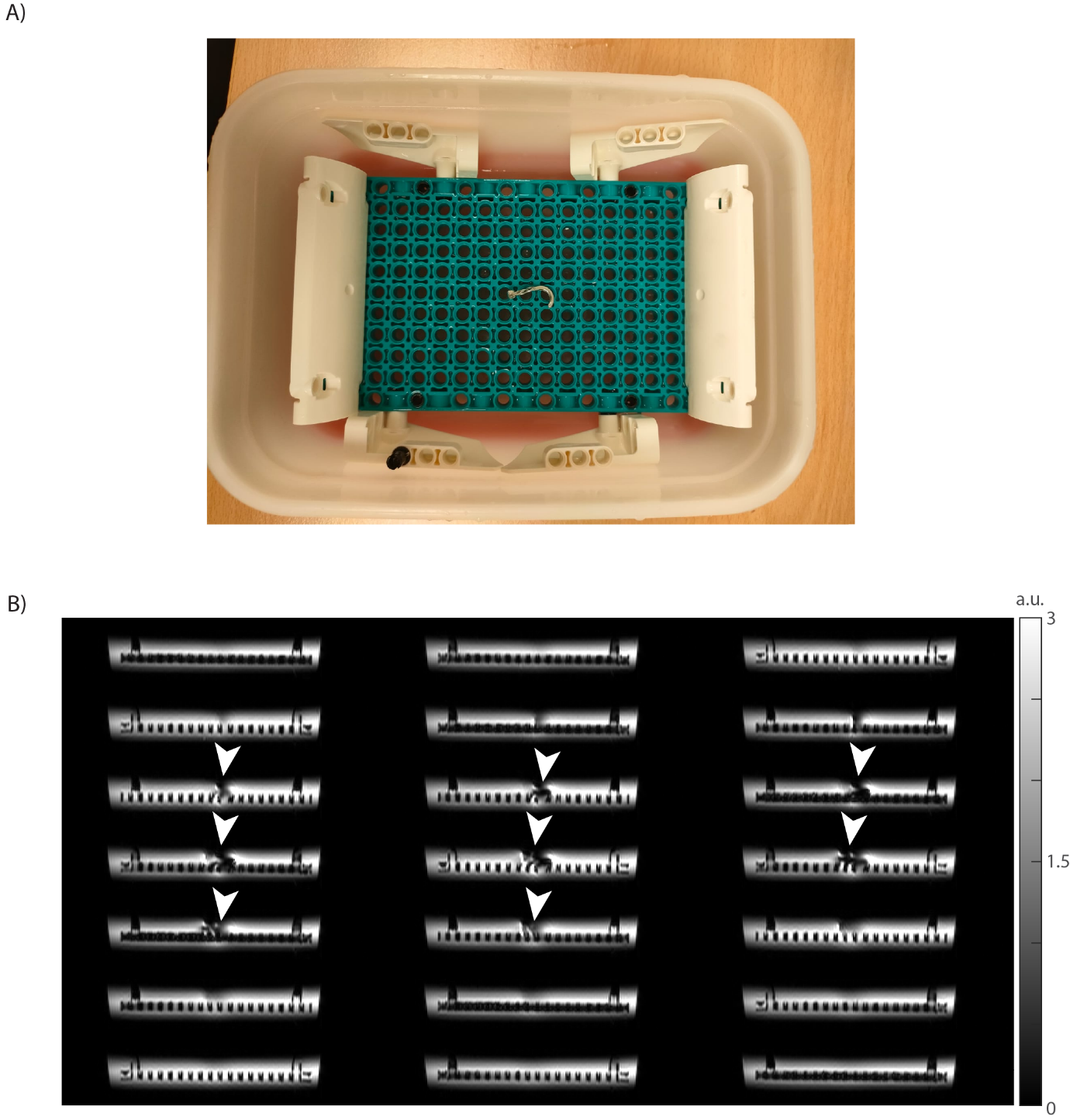


Supporting Information Figure S1. A) Titanium aneurysm clip (2 cm), used in the phantom and in-vivo scans, placed on the phantom; a plastic grid with 8.0 mm periodicity. B) Reformat demonstrating the distortion of the slice profile, indicated by white arrowheads, of the phantom as shown in A). Images were to demonstrate the through-plane effect.


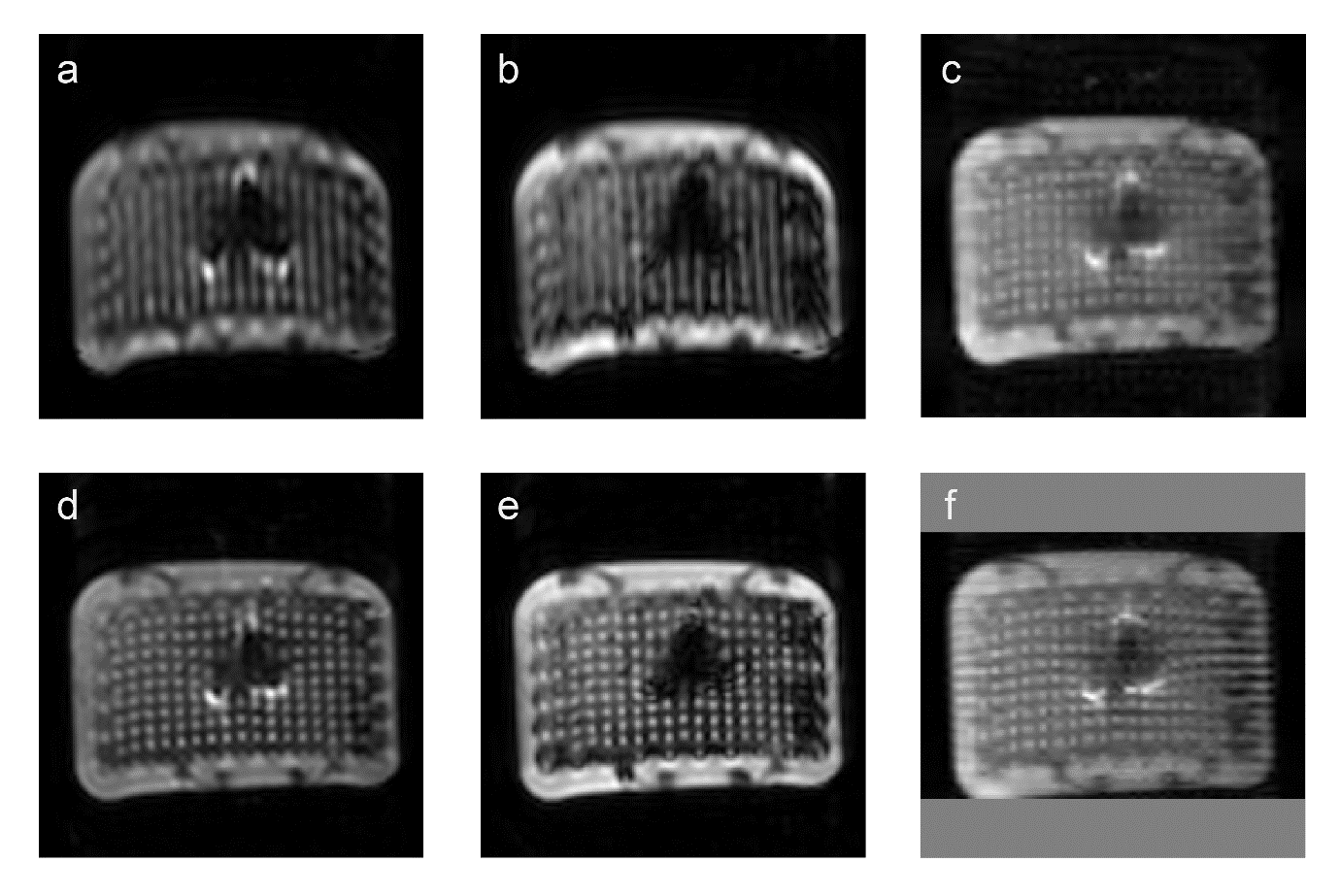


Supporting Information Figure S2. Images of a phantom grid with aneurysm clip, showing SE-EPI (a,d), GE-EPI (b,e) and SPEN (c,f) at comparable acquired base resolutions $\Delta y_{SPEN}$ and $\Delta y_{EPI}$. SPEN images were acquired using Q=75. Top row: Δy = 5.7 mm; bottom row Δy = 3.1 mm. To achieve 3.1 mm base resolution with SPEN, the FOV was reduced to 140 mm. Images demonstrate the improved resolution when a closer linespacing $\Delta y_{SPEN}$ is used in acquisition.


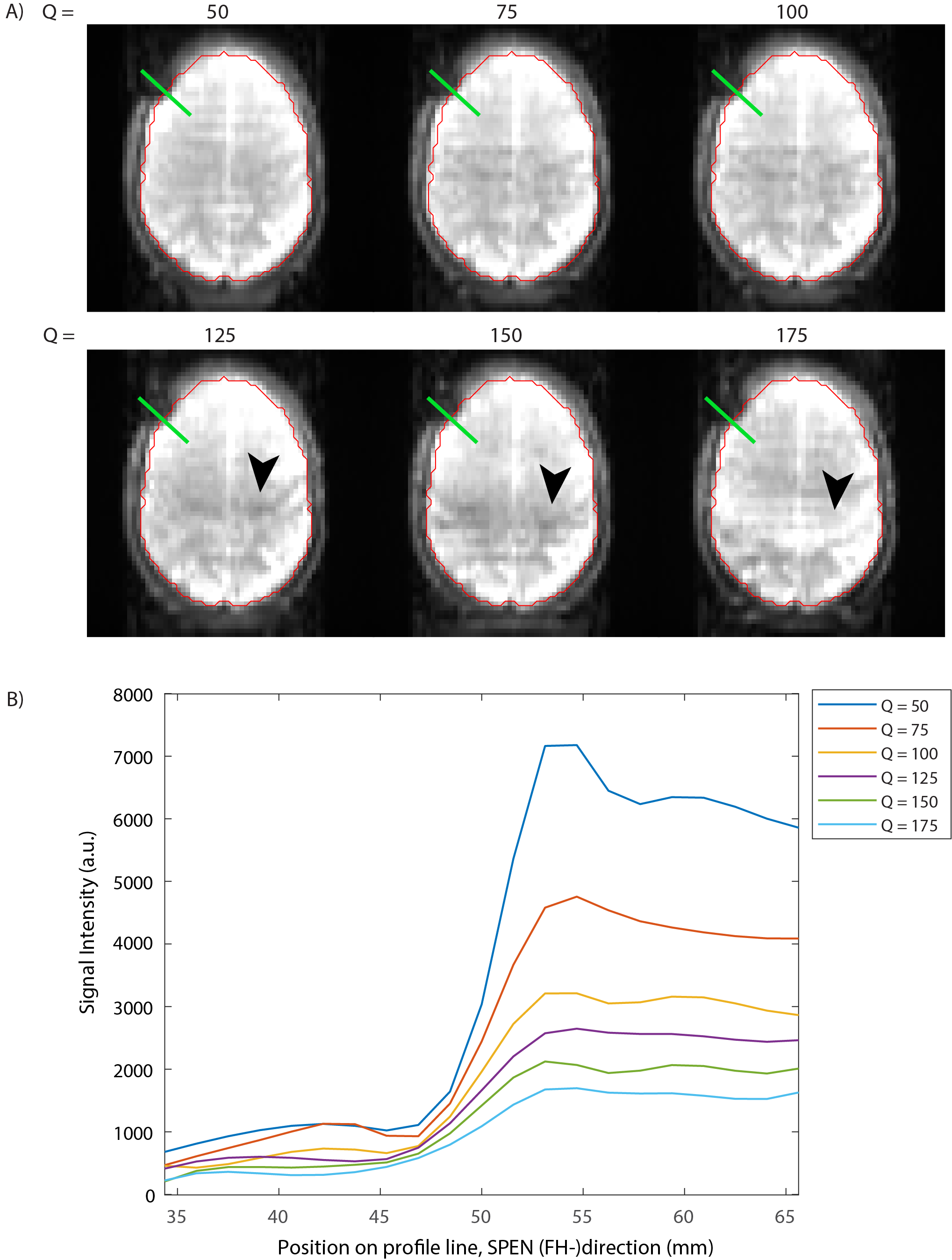


Supporting Information Figure S3. SPEN label-images for Q-values ranging between 50 and 175. A) SPEN Label images, edge artifacts are visible for higher Q-values as a result of too few measurement points, indicated by black arrowheads B) Intensity profiles along the green line in A, for all Q-values, using a spline interpolation with a factor of 2. The intensity step remains roughly at the same location for the different Q-values.

**Supporting Information**

**SPEN encoding and deformation**

In SPEN imaging, the key determinant of spatial deformation in SPEN direction, is the time averaged gradient strength $G_{SPEN}$ in SPEN direction, much like the time averaged gradient strength $G_{EPI}$ in phase encoding direction determines the spatial deformation in EPI encoding. The natural quantity and unit to express the sensitivity to off resonance in is the bandwidth, BW, of the sequence in Hz per m, thus $BW=\frac{\gamma}{2\pi}G$.

If we observe the condition that |*G_exc_T_exc_*| = |*G_acq_T_acq_*| and if we operate in fully refocused mode, such that $T_{exc}= T_{acq}$, and correspondingly $G_{exc}= G_{acq}\equiv G_{SPEN}$, the degrees of freedom in the sequence are reduced, and the sought after bandwidth is simply $BW_{ENC}(\frac{Hz}{m})={\frac{\gamma}{2\pi}G}_{SPEN}$.

The literature on SPEN has introduced 2 additional parameters that could be considered useful for controlling the off resonance sensitivity of a SPEN acquisition. In Ben-Eliezer et al. (2010), the sweep rate of the chirp pulse (given in rad/s^2^) $R=\frac{2\pi BW_{chirp}}{T_{exc}}$ , with BW_chirp_ in Hz, has a clear link with $BW_{ENC}$ of the sequence, since via the relations imposed by full refocusing, we have:

$$BW_{ENC}(\frac{Hz}{m})={\frac{\gamma}{2\pi}G}_{SPEN}=\frac{R}{2\pi}\frac{T_{exc}}{L_{pe}}$$

This relation naturally arises from the fact that R is also directly derived from a bandwidth.

R also determines the sharpness of the quadratic phase profile $\varphi\left( y \right)=\alpha y^{2}+\beta y+c$ that is imparted on the spin system after excitation, with

$$\alpha=-\gamma^{2}G_{exc}^{2}/2R$$

R is thus a key determinant of the resolution, also before applying super resolution reconstruction. (The factor 2 here arises since Ben Eliezer applied the sweep on the 90 degree excitation pulse)

In Liberman et al. (2018) for example, the Q parameter used in this paper is introduced. Q is the time bandwidth product of the chirp pulse, and as such has a similarly natural relation with spatial deformation and $BW_{ENC}$. In this paper, first the relation with resolution was developed by using

$$\alpha=-\frac{Q}{L_{pe}^{2}}$$

whilst focusing less on its bandwidth properties:

$$Q=2\pi\mathrm{BW}_{\mathrm{chirp}}T_{exc}= \gamma G_{exc}L_{pe} T_{exc}$$

from which it follows that:

$$BW_{ENC}\left( \frac{Hz}{m} \right)={\frac{\gamma}{2\pi}G}_{SPEN}=\frac{Q}{2\pi L_{pe} T_{exc}}$$

which clearly brings forward the expected role of Q for controlling geometrical distortions, given that the field of view and $T_{exc}$ remain unchanged.
